# Notes on Syphilis

**Published:** 1874-01-01

**Authors:** 


### Original Translations.

### NOTES ON SYPHILIS.

*Translated for* THE EXAMINER, *from La France Medicale of Nov. 5th, 12th, and 15th.*

LANCEREAUX on the treatment of acquired syphilis (*La France Medicale*, Nov. 5, 1873): 1. Period of primary accident, or syphilitic chancre: No mercury should be employed; the regime should be slightly tonic, ferruginous, and hydrotherapeutic. Mercury is to be exhibited only in order to procure resolution of indurations which are indolent in disappearing, and is the more useful and necessary as the ganglionic system is more profoundly affected. In all other cases observe scrupulous cleanliness, and employ lotions of aromatic wine, or alcohol mixed with a variable quantity of water; and dress simply with calomel ointment, or dry lint. Cauterization is useless, if not dangerous.

2. Period of secondary accidents: When local manifestations, though imminent, are not yet declared, and the patient is in a prodromic stage, we find intense cephalalgia, general lassitude, wandering pains, and moral depression. These point to a speedy explosion of the disease. Mercury is not yet indicated, but laxatives, if the secretions are disturbed; iron, if there be chloro-anæmia; and, as a rule, rest, by the aid of opium and baths. Mercury is to be exhibited when exanthemata occur, even if these be of the roseolous and papular varieties, for which M. Diday considers such a medication valueless. Discontinue the mineral on subsidence of the eruption, lest anæmia or *obesity* (sic.) be induced. The author does not recommend the administration of mercury by inunction, or subcutaneous injection, but prefers the use of the bichloride or protochloride internally, especially the former, and discontinues the remedy on the disappearance of all syphilitic manifestations. The intercurrent indications are to be met. Baths, and the iodide of potassium, are useful in case of syphilitic fever, and at the outset of secondary manifestations.

3. Period of tertiary accidents: Iodide of potassium is generally indicated. In cases of visceral complication, calomel, in minute doses, should be tried. In some intestinal derangements, due to lesion of the hæmatopoetic glands (liver and amyloid spleen), mercurial friction is recommended, and the use of nitric lemonade (Bud). Ulcers of foul aspect require glycerine, alcohol, tincture of iodine, sulphate of copper, nitric acid, perchloride of iron, corrosive sublimate, iodoform, more or less concentrated solution of chloral, or meta-chloral (Duj.-Beaumetz). Syphilitic arthropathy, osteocopic pain, and osteoperiostitis, require vesication. For rebellious exostoses apply vesicatories, and dress with tincture of iodine or Neapolitan ointment and emollient cataplasms.

*La France Medicale*, Nov. 15, 1873: Syphilitic dermatosis cured by Lewin's treatment. N. had treated a blenorrhagia, by imbibition of a decoction of sarsaparilla, without effect. The discharge finally ceased on his remaining for hours in a rain-storm, when his clothes became thoroughly wet. Soon after, a crustaceous eruption appeared on the scalp, which was diagnosticated as impetiginous eczema. Preparations of sulphur aggravated the disorder, and the use of the sulphurous thermal baths of Grabalos, in Spain, brought about a slight diminution of the eruption upon the scalp, and its general appearance upon the surface of the body, especially upon the right leg, from knee to foot. Three additional resorts to thermal treatment, under the direction of as many different physcians, resulted in his confinement to bed. Dr. Badia, who was then summoned, established the existence of alopecia, induced by non-catarrhal eczema, catarrhal (sero-purulent) eczema, and impetigo of the right thigh, patches of serpiginous impetigo in the flexures of the joints, light and bronze-yellow maculæ generally, hyperidrosis of integument, especially on the inner faces of the thighs, and, lastly, acute varicella, pursuing its usual course. In making a syphilitic diagnosis, Dr. Badia was influenced by a belief that sulphurous remedies and sulphur-waters constituted a touch-stone for syphilitic dermatoses, since their influence was negative in a curative direction, and produced a positive aggravation of symptoms. The color and peculiar grouping of the maculæ, the absence of itching, and the discovery of a syphilitic taint in the wife and children of the patient, confirmed the opinion. In accordance with the therapeutic method of Prof. Lewin, of Berlin, a subcutaneous injection of a solution of the bichloride of mercury was employed daily, the amount injected being increased every fortnight, while topical applications were made with phenic acid and mercurial ointments.

*La France Medicale*, Nov. 12, 1873, contains a review of a recent publication by Dr. Corlieu on the mortality of the royal families of France. In a pathological point of view, no greater interest attaches to the diseases of a royal family than to those of the commonest of their subjects. A discussion of the question whether the house of Valois had a syphilitic taint may be of interest to students of history in general, and the French nation in particular, but hardly presents an attraction for scientists. There is, however, one point in the determination of the facts of the case, which has a bearing on the genetic relations of syphilis to morbid diathesis. Roubaud gives the following details:

"Francis I., crowned at twenty-one years of age, contracted a venereal disease, either before or after the birth of his children. The first son, Francis, died when nineteen years old, and is said to have been poisoned by Montecuculli. Charles, the second son, died at twenty-nine years of age, in Picardy, of the plague. Henry II., the youngest son, was killed, in his forty-first year, in a tournament. The first daughter, Louise, died when two years old; the second, Charlotte, in her eighth year; the third, Madeleine, wife of the King of Spain, died in her seventeenth year, of fever; while Margaret, the youngest daughter, survived till her fifty-first year, and became the wife of the Duke of Saxony."

The children of Henry II. are cited in proof of the transmission of the taint, and the genesis of a diathesis. "The first son, Francis II., died childless, of scrofulous caries of the ear, at seventeen years of age; the second, Louis d'Orleans, died of scrofula, in his second year; the third, Charles IX., died of phthisis, aged twenty-four; while the fourth, Henry III., was assassinated in his thirty-eighth year. The first daughter, Elizabeth of France, died at twenty-three years of age; the second, Claude, at twenty-seven years; the third and fourth, twins, died almost at birth; and the last, Margaret of Navarre, lived to be sixty-two years old."

Per contra, it may be remarked:

First. The fact of the *syphilitic* infection of Francis I. is not admitted by all historians. The story related by L. Guyon (*Lecons Diverses* No. ii.) though accepted by Mezerai, Garnier, Gaillard, and others, is not universally credited. He is there stated to have had criminal relations with the wife of a barrister named Feron, who, in order to revenge himself upon the King, contracted a venereal disorder in a notorious resort, and succeeded in infecting his wife (who subsequently died of the disease), and through her the royal seducer. But Francis I. was sick in Compiegne, in 1538, in consequence of some disorder of the genito-urinary organs, from which he is said to have suffered for eight years previously; while it is known that, in 1519, Leonardo da Vinci died, who painted the portrait which tradition has named *La Belle Feronniere*, and which represents a lovely woman in the bloom of healthful beauty. (See Martin's voluminous History of the Kings of France, vol. viii., Paris, 1844.)

Second. The *nature* of the disease incurred by Francis I. has not been accurately determined. He died of exhaustion and irritative fever, occasioned by what has been called an "inveterate ulcer." But it has been definitely determined that that "ulcer" was, in reality, a vesico-perineal fistula. Such a lesion certainly does not point to syphilitic antecedents, but rather to a chain of events commencing with a urethral stricture, possibly of blenorrhagic origin, and continuing with the occurrence of urethral abscess, and more or less cystitis at the bas fond of the bladder. Such a complication would be serious enough for the surgery of the sixteenth century. Gaillard (vol. vii., p. 355) states that the King was partially relieved by a Jewish physician, who placed him upon a diet of asses' milk.

Third. It is merely shown that, of eighteen individuals in one family, two died of scrofula and one of pulmonary consumption, a truly small proportion in view of the excesses and debaucheries of the race and of the period. Nor can the complete extinction of the house of Valois, brought about by unfruitful marriages, be adduced in proof of hereditary taint, since consanguinity in marriage is almost a necessity in royal families, and would tend to produce such results: while the frequency of hereditary syphilis is evidence that sterility and impotency are not the natural agencies by which the disease is self-limited.

It is fair to conclude, then, that the dyscrasia evident in the descendants of Henry II. is not *proven* to have originated in a syphilitic taint of the grandfather. And it is worthy of note that Charles IX., whose last words, almost inaudible on account of a pulmonary hæmorrhage, bore witness to his satisfaction that he left no male infant to wear the crown after him, was yet the father of a healthy son. That son was born of his mistress, Marie Touchet, and he arrived at adult years to the dukedom of D'Angouleme.

